# Isolation and Characterization of the Flavonol Regulator CcMYB12 From the Globe Artichoke [*Cynara cardunculus* var. *scolymus* (L.) Fiori]

**DOI:** 10.3389/fpls.2018.00941

**Published:** 2018-07-04

**Authors:** Emanuela Blanco, Wilma Sabetta, Donatella Danzi, Donatella Negro, Valentina Passeri, Antonino De Lisi, Francesco Paolocci, Gabriella Sonnante

**Affiliations:** ^1^Institute of Biosciences and Bioresources, National Research Council, Bari, Italy; ^2^Institute of Biosciences and Bioresources, National Research Council, Perugia, Italy

**Keywords:** artichoke, flavonoid biosynthesis, flavonol, flower color, healthy compounds, qRT-PCR, R2R3-MYB, transcription factor

## Abstract

Flavonoids are a well-studied group of secondary metabolites, belonging to the phenylpropanoid pathway. Flavonoids are known to exhibit health promoting effects such as antioxidant capacities, anti-cancer and anti-inflammatory activity. Globe artichoke is an important source of bioactive phenolic compounds, including flavonoids. To study the regulation of their biosynthesis, a *R2R3-MYB* transcription factor, *CcMYB12*, was isolated from artichoke leaves. Phylogenetic analysis showed that this protein belongs to the MYB subgroup 7 (flavonol-specific MYB), which includes Arabidopsis AtMYB12, grapevine VvMYBF1, and tomato SlMYB12. *CcMYB12* transcripts were detected specifically in artichoke immature inflorescence and young leaves and overlapped with the profiles of flavonol biosynthetic genes. Electrophoretic mobility shift assays (EMSAs) revealed that recombinant CcMYB12 protein is able to bind to ACII element, a DNA binding site ubiquitously present in the promoters of genes encoding flavonol biosynthetic enzymes. In transgenic Arabidopsis plants, the overexpression of *CcMYB12* activated the expression of endogenous flavonol biosynthesis genes, leading to an increase of flavonol accumulation and a decrease of anthocyanins in leaves. Likewise, in transgenic tobacco petals and leaves, the overexpression of *CcMYB12* decreased anthocyanin levels and increased flavonols.

## Highlights

The artichoke *CcMYB12* is expressed in flavonol-rich organs. Its overexpression in Arabidopsis and tobacco causes an activation of early flavonoid biosynthetic genes and a metabolic flux diversion from anthocyanins to flavonols.

## Introduction

In the last decades, an increasing attention has been paid to nutrition and its effects on the development of chronic degenerative diseases. The correct nutritional habits in a population can act as important factors in primary and secondary levels of prevention, thus reducing health risks (Sofi et al., 2013). The Mediterranean diet, recognized to be protective against the occurrence of numerous diseases, is characterized by a high consumption of plant-based food, such as legumes, cereals, olive oil, fruits and vegetables, and many of these plants contain functional components with positive effects on health (Ortega, 2006).

Several fruits and vegetables are especially rich in polyphenols, bioactive secondary metabolites, which constitute a highly diverse group with an extremely large structural diversity (flavonoids, phenolic acids, hydroxycinnamic acid derivatives, lignans). In particular, flavonoids are considered as health-protective components as they are able to protect against cardiovascular diseases, some types of cancers (Ravishankar et al., 2013), degenerative disorders (Korkina, 2007), platelet aggregation (Graf et al., 2005), and osteoporosis (Trivedi et al., 2009).

The globe artichoke [*Cynara cardunculus* var. *scolymus* (L.) Fiori] represents a relevant constituent of the Mediterranean diet (Gatto et al., 2013), as it is a natural functional food rich in bioactive polyphenolic compounds, inulin, fibers and minerals. Nutritional and pharmaceutical properties of the edible part of artichoke, its immature inflorescences, also called capitula or heads (receptacles with inner and intermediate bracts), can be ascribed to high levels of health-promoting polyphenolic compounds, such as caffeoylquinic derivatives and flavonoids (Sonnante et al., 2010), known to have a marked antioxidant activity (Rice-Evans et al., 1997; Azzini et al., 2007). Moreover, artichoke leaf extracts are known to possess antioxidative, anticarcinogenic, anti immunodeficiency virus, cholesterol-lowering, bile-expelling, and hepatoprotective activities, along with antifungal and antibacterial properties (Agarwal and Mukhtar, 1996; Gebhardt, 1997; Kraft, 1997; Brown and Rice-Evans, 1998; McDougall et al., 1998; Shimoda et al., 2003; Zhua et al., 2005).

The flavonoid biosynthetic pathway is well-established, the structural genes of the enzymes involved in flavonoid formation being mainly regulated at the transcription level (Ververidis et al., 2007; Liu et al., 2015). Transcription factors (TFs) usually constitute gene families and regulate target genes in a tissue- and species-related manner, and/or in response to various biotic and abiotic stress factors (Bovy et al., 2002; Koes et al., 2005; Xu et al., 2015). TFs regulating metabolic networks have been considered as promising tools for engineering the levels of metabolites (Grotewold, 2008). Moreover, the association analyses between the expression of TFs and metabolic profiles could lead to the selection and promotion of specific genotypes with a healthier metabolite accumulation and better stress tolerance.

Flavonoid transcriptional regulators mostly belong to protein families containing R2R3-MYB domains, basic helix-loop-helix (bHLH) domains (MYC proteins), and conserved WD amino acids repeats (Ververidis et al., 2007). *ZmP1* from maize was the first MYB factor, involved in flavonoids accumulation, displaying activity without binding a bHLH protein (Grotewold et al., 1994). The Arabidopsis ortholog, *AtMYB12*, was isolated and fully characterized as flavonol-specific regulator (Mehrtens et al., 2005; Stracke et al., 2007). It was heterologously expressed in plants, like tobacco and tomato, producing high-level accumulation of polyphenolic compounds, particularly flavonols and chlorogenic acid (CGA; Luo et al., 2008).

Flavonoid composition among plant species can be highly different and TF activity varies depending on the investigated plant, highlighting the importance of determining the specificity of TF for their target genes in various species (Luo et al., 2008; Czemmel et al., 2017). The role of MYB12 homologues in flavonoid regulation has been established in many plant species, e.g., grapevine, tomato and Japanese gentian (Czemmel et al., 2009; Ballester et al., 2010; Nakatsuka et al., 2012; Czemmel et al., 2017).

Despite the known beneficial properties of phenylpropanoids from artichoke, their biosynthetic routes in this crop are not well defined yet. The biosynthesis of chlorogenic acid and its derivatives was recently studied (Comino et al., 2007, 2009; De Paolis et al., 2008; Moglia et al., 2009; Sonnante et al., 2010), whereas more recently, a flavonoid 3′-hydroxylase, *CcF3′H*, implicated in dihydroxylated flavone and flavonol biosynthesis, and in anthocyanin accumulation, was identified (De Palma et al., 2014). To shed light on the accumulation of beneficial compounds in artichoke, regulatory mechanisms of flavonoid synthesis need to be examined.

To this end, here we report on the isolation and functional characterization of *CcMYB12*, the artichoke putative homologue of R2R3-MYBs controlling the biosynthesis of flavonols in a number of species. We show that CcMYB12 recombinant protein can bind canonical AC element present on the promoters of artichoke early flavonoid genes and that its transcript expression profile overlaps with those of several early flavonoid genes in flavonol- rich artichoke bracts. Finally, by targeted metabolic and molecular analyses we show that the ectopic expression of *CcMYB12* in Arabidopsis and tobacco plants promotes flavonol biosynthesis at expenses of anthocyanins.

## Materials and Methods

### Plant Materials

Plants of globe artichoke (cv. Mola) were grown in the experimental field of the Institute of Biosciences and Bioresources (IBBR-CNR), Bari, Italy. Material from three independent plants was collected from stems, young and adult leaves; flower heads were harvested at the commercial maturity stage and separated into external, intermediate, internal bracts and receptacle (Sonnante et al., 2010).

Arabidopsis (*Arabidopsis thaliana* ecotype Columbia [Col-0]) seeds were germinated on Murashige and Skoog plates (Murashige and Skoog, 1962) containing 1% sucrose in a growth chamber (250 μmol photons m^-2^ s^-1^; 22°C) under a long-day regime (16 h of light/8 h of dark). To study the gene expression and metabolite profiles in WT and CcMYB12 transgenic Arabidopsis leaves, seedlings were transferred to soil in a climate-controlled chamber under the same photoperiod and leaves collected 4 weeks after germination. To analyze the effects of sucrose on anthocyanins accumulation in the seedlings, WT and CcMYB12 transgenic seeds were germinated *in vitro* either on 1% or 6% (w/v) sucrose concentration and 2-week old seedlings collected for molecular and metabolic analyses. *Nicotiana tabacum* cv. ‘SR-1’ plants were grown *in vitro* on Murashige and Skoog solid medium supplemented with 3% (w/v) Suc at 23°C and 16/8-h light/dark photoperiod for rooting and then transferred to soil with the same environmental conditions.

### Full-Length cDNA and Promoter Sequence Isolation *of CcMYB12*

Total RNA was isolated from 0.2 g of frozen young leaf of artichoke using RNeasy Plant Mini Kit (QIAGEN, Hilden, Germany). cDNA was synthesized from 1 μg of total RNA using the QuantiTect Reverse Transcription Kit (QIAGEN).

The NCBI^1^ globe artichoke EST database was screened to find *AtMYB12* homologues (Accession No. CAB09172; Stracke et al., 2001). On the retrieved sequence the primer pair MYB12F1-MYB12R1 (Supplementary Table S1) was designed and employed to amplify a partial cDNA sequence from leaves. Amplification reactions contained 1 μL of cDNA, 1X PCR buffer, 0.4 mM deoxyribonucleotide triphosphates (dNTPs), 400 nM each primer, and 1 unit of JumpStart Taq DNA Polymerase (Sigma–Aldrich, Saint Louis, MO, United States), in a final volume of 25 μl. The thermal cycling program included a step at 94°C for 3 min, followed by 35 cycles of 94°C for 30 s, 55°C for 30 s, and 72°C for 1 min, and a final extension at 72°C for 10 min. PCR fragments were cloned using the GENEJet PCR Cloning kit (Thermo Fisher Scientific, Waltham, MA United States) and subsequently sequenced. New primers pair designed on the resulting amplicon were then employed to clone the 5′ and 3′ *CcMYB12* ends (Supplementary Table S1) with the 5′ and 3′ RACE kits, respectively (Invitrogen, Thermo Fisher Scientific, Waltham, MA United States). Then, the complete *CcMYB12* open reading frame (GenBank Accession No. MG517449) was PCR amplified (CcMyb12-FWatg - CcMyb12-RVstop, Supplementary Table S1) and confirmed by sequencing.

The GenomeWalker Universal kit (Clontech Laboratories, Inc. United States) was used to isolate the *CcMYB12* promoter (GenBank Accession No. MG517450), following manufacturer’s instructions. After artichoke genomic DNA digestion with *DraI*, *EcoRV*, *PvuII*, and *StuI*, and ligation to adaptors, nested PCRs were performed for each digested-ligated DNA library, using the supplied AP1 primer from the kit and *CcMYB12* specific primers (GWCcMYB12_GSP1 and GWCcMYB12_GSP2; Supplementary Table S1). Amplicons were resolved on a low-melting point agarose gel, and fragments longer than 1 kb were excised from the gel, purified, cloned, and sequenced.

The *CcMYB12* full-length cDNA was analyzed by NCBI BLAST^2^. For the multiple sequence alignment analysis, the amino acid sequence of CcMYB12 and other MYB homologs from different plant species retrieved from NCBI were aligned using Clustal Omega^3^, Jalview v. 2.10.1 (Waterhouse et al., 2009) and Bioedit v. 7.2.5 softwares^4^. A phylogenetic tree was constructed using the neighbor-joining method with the minimum evolution test and p-distance model with 10,000 bootstrap replicates, using the MEGA package v. 6.^5^ The theoretical molecular weight and isoelectronic point (pI) of CcMYB12 protein were calculated using ProtParam tool.^6^ The conserved domain of CcMYB12 protein was scanned by the InterProScan program^7^. PlantPAN Interface^8^ and PlantCARE^9^ were used to analyze promoter sequence.

### Production of Stable Arabidopsis and Tobacco *CcMYB12* Transformants

The full length coding sequence of *CcMYB12* was amplified using specific primers (GW-MYB12-F and CcMYB12-RVstop, Supplementary Table S1). *CcMYB12* was firstly cloned into the entry vector pENTR/D-TOPO (Invitrogen) using the Gateway recombination system, and then recombined into the destination vectors pK2GW7 (Karimi et al., 2002) between the cauliflower mosaic virus 35S promoter and the t-nos terminator construct, and pMDC32 (Curtis and Grossniklaus, 2003), between a double mosaic virus 35S promoter and the t-nos terminator. In both cassettes the cDNA insertion was proven to be in the sense orientation by sequencing. pK2GWF7-*CcMYB12* and pMDC32-*CcMYB12* plasmids were introduced into *Agrobacterium tumefaciens* strains GV3101, and LBA4404, respectively, by the freeze-thaw method (Hofgen and Willmitzer, 1988).

The recombinant *A. tumefaciens* GV3101 strain harboring either the pK2GW7-*CcMYB12* plasmid or the empty vector were used to stably transform Arabidopsis plants according to the floral dip method (Clough and Bent, 1998), and the *A. tumefaciens* LBA4404 strain harboring the pMDC32-*CcMYB12* plasmid was used to stably transform leaf disks of *N. tabacum* cv SR1 according to Horsch et al. (1985).

To select Arabidopsis transgenic plants, T_1_ seeds were allowed to germinate on selected Murashige and Skoog medium (Murashige and Skoog, 1962) containing 1% Sucrose and 50 mg/l kanamycin at 250 μmol photons m^-2^ s^-1^ and 22°C. Stable insertion of the exogenous expression cassette was checked by genomic DNA amplification with primers for the neomycin phosphotransferase (*nptII*) gene (Supplementary Table S1) and for the *CcMYB12* cDNA sequence (MYB1_F and CcMyb12-RVstop; Supplementary Table S1).

Transgenic tobacco lines were *in vitro* selected on MS medium supplemented with 20 mg/L hygromycin. Hygromycin-resistant tobacco plantlets were recovered and screened by PCR for the presence of *CcMYB12* and *hptII* genes (Supplementary Table S1) and then moved to glasshouse under outdoor environmental conditions. As control, *CcMYB12* primary transgenic tobacco plants (T_1_) were grown side by side in the same glasshouse with SR1 untransformed plants. Seeds from self-pollinated tobacco transgenic T_1_, and control lines were collected, surface sterilized, plated onto MS medium and then grown in the glasshouse as reported above.

Leaves from tobacco and Arabidopsis, and tobacco flowers from T_2_ and T_3_ plants were used for molecular and metabolic analyses.

### Quantitative Real-Time PCR in Artichoke, Transgenic Arabidopsis and Tobacco Plants

Total RNA was isolated from artichoke as described above. Total RNA of wild type (WT), transgenic Arabidopsis leaves and seedlings and tobacco leaves and petals of fully opened flowers, at stage 12 as reported in Koltunow et al. (1990), was isolated using RNeasy Plant Mini Kit (Qiagen). cDNA was synthesized from 1 μg of total RNA using the QuantiTect Reverse Transcription Kit (Qiagen) according to the manufacturer’s instructions. Primers for quantitative Real-Time PCR (qRT-PCR) were designed on *CcMYB12*, or on known gene sequences retrieved from NCBI database or taken from the literature (Supplementary Table S2). As for putative artichoke flavonol synthase (*FLS*), the NCBI *Cynara* EST database was screened using the putative *Helianthus annuus FLS* coding sequence (XM_022126949.1) as query. The artichoke GE604603.1 sequence, showing the highest similarity, was retrieved and primers therein designed (Supplementary Table S2).

Reactions were performed on a CFX96 Touch Real-Time PCR Detection System (Bio-Rad, United States), using iTaq Universal SYBR Green Supermix (Bio-Rad). Reaction mixtures (10 μl) included the following components: 1Xmaster mix, 0.2 μM of each primer and 1 μl cDNA. Cycle conditions were 95°C for 20 s and then 40 cycles of 95°C for 15 s and 60°C for 30 s. Normalization against different reference genes was tested prior to qRT-PCR analyses. Actin and Elongation Factor1α (*EF1*α) genes expression were analyzed for both tobacco and Arabidopsis experiments, in addition tobacco tubulin expression was tested. The expression levels of each gene were normalized by the expression of actin genes both in Arabidopsis and tobacco (*At3g18780* and AF154640, respectively). The artichoke *EF1*α gene (EU442190) was used as reference gene in artichoke analyses, as previously tested (Sonnante et al., 2010). Separation of real-time PCR products on 2% (w/v) agarose gels revealed single bands of the expected size whose identities were confirmed by direct sequencing. Relative quantification was performed according to the comparative Ct (threshold cycle) method (2^-ΔΔCt^; Bustin, 2000; Palmieri et al., 2008). In all experiments, appropriate negative controls containing no template were used. Analysis was performed on three independent biological replicates per plant sample, and three technical replicates for each sample, and reactions were repeated twice to verify reproducibility.

### Heterologous Expression of Recombinant CcMYB12 in *Escherichia coli* and Electrophoretic Mobility Shift Assay

CcMYB12 recombinant protein in frame with a C-terminal (6XHis)-tag was expressed in *E. coli* BL-21 Rosetta cells. The coding sequence without the terminal codon was PCR amplified from cDNA of artichoke young leaf (GW-MYB12-F and pE-MYB12-R; Supplementary Table S1) and cloned into the pENTR^TM^/D-TOPO^R^ Vector (cat. No. K2400-20, Invitrogen, United States) using the Gateway recombination system. The insert was sequenced to confirm identity. Subsequently, the CcMYB12 entry clone was used to generate the expression clone by a recombination reaction with the pET-DEST42 Gateway^TM^ destination vector, encoding a C-terminal (6XHis)-tag, under the control of the Cauliflower Mosaic Virus 35S promoter (Cat. No. 12276-010, Invitrogen, United States). The insert was sequenced prior to protein expression. Growth of *E. coli* expression cultures in selective liquid LB (100 μg/ml ampicillin) and protein induction were performed as described in the QIAexpressionist manual (protocol 7; QIAGEN, Germany).

Recombinant protein was batch-purified by affinity chromatography using a Ni-NTA resin under denaturing conditions according to instructions (QIAexpressionist, Protocol 10; QIAGEN, Germany) with the exception that lysis buffer B added with imidazole (100 mM NaH_2_PO_4_; 10 mM Tris⋅Cl; 8 M urea; 10 mM Imidazole, pH to 8.0 using NaOH) was used to prepare cleared *E. coli* lysates. The eluted fractions were collected, concentrated with 3 volumes of 100% acetone and re-suspended in Phosphate Buffered Saline pH 7.4. Total protein content from crude extracts and eluted fractions was quantified by Bradford Protein Assay (Bio-Rad, United States) (Bradford, 1976), while purification efficiency was verified by SDS-PAGE (Laemmli, 1970) and Coomassie Blue staining. *E. coli* extracts harboring no vector/plasmid were used as negative control.

To perform electrophoretic mobility shift assays (EMSA), the AC-rich sequences ACI and ACII containing the MYB-binding *cis*-motifs ACCAACC and ACCTACC, respectively, were used as MYB-binding sites in two different probes (Patzlaff et al., 2003; Xu et al., 2013). Related mutated probes (mACI and mACII) were used as negative controls. The oligonucleotide probes ACI (gatcctttatacccACCTACCagacacg) mACI (gatcctttatacccCAAGCAAagacacg), ACII (gatccttctccACCAACCcccttcacttcccg) and mACII (gatccttctccGAAGGAAcccttcacttcccg) were labeled with Biotin-11-UTP onto the 3′ end using the Pierce Biotin 3′-End DNA Labeling Kit (Life Technologies), following manufactory’s instructions. About 0.6 μg purified recombinant CcMYB12 protein was allowed to re-nature prior to the binding experiment. Each binding assay was carried out using the LightShift Chemiluminescent EMSA Kit (Thermo Scientific, United States), with few modifications to the general instructions: the incubation time for binding reaction was extended up to 60 min, while the incubation time of membrane with the substrate working solution was reduced to 2 min. The bound complexes were subjected to electrophoresis on native 7% polyacrylamide gels in 0.5% Tris–borate EDTA (TBE) buffer (pH 8.0) at 90 V for 60–80 min at 4°C, and then transferred to a positively charged nylon membrane (Biodyne B Nylon Membrane for Chemiluminescent EMSA, Thermo Scientific) using the Trans-Blot^®^ SD Semi-Dry Transfer Cell (Bio-Rad). The biotin-labeled signals were detected using the Streptavidin-Horseradish Peroxidase Conjugate, the chemiluminescent substrate and the chemilumino-image analyzer Chemi Doc (Bio-Rad).

### Flavonoid and Monocaffeoylquinic Acid Analyses in Transgenic Arabidopsis and Tobacco Plants

Flavonoids were extracted from freeze-dried samples with 40 μL of 70% MeOH per mg of dry weight (DW) of tissue. Extracts were centrifuged at 16,000 × g and supernatants were filter spun using 0.2 μm filters analyses. HPLC analyses were carried out with a Beckman-Coulter (Fullerton, CA, United States) HPLC System Gold chromatograph equipped with a DAD programmable detector (System Gold, series 166) operated by a 32 Karat software package (Beckman-Coulter).

Arabidopsis flavonols were analyzed by the HPLC method proposed by Mehrtens et al. (2005), modified by Negro et al. (2012) using the reversed phase column Luna C18 (250 × 4.6 mm i.d., particle size 5 μm; Phenomenex, Torrance, CA, United States). Diode array detection was between 200 and 600 nm, and absorbance was recorded at 280, 310, and 350 nm. Peaks were classified as corresponding to kaempferol or quercetin derivatives by UV spectral analysis.

HPLC analyses of tobacco flavonoids and monocaffeoylquinic acids and Arabidopsis anthocyanins were carried out as described in Luo et al. (2007), using a Synergi 4 μm Hydro-RP 80A column (250 × 4,60 mm internal diameter; Phenomenex, Torrance, CA, United States). Anthocyanin contents were calculated from their peak areas at 520 nm in the chromatograms and compared with the external standard cyanidin chloride; flavonoid absorbance was recorded at 325 and 520 nm.

Flavonoid and monocaffeoylquinic acid identification was reached by combining the following information: positions of absorption maxima (λ max) and retention times (min) were compared with those from pure standards and/or interpreted with the help of structural models already available in the literature, the latter identities considered as probable rather than proven. Quantification was made by using six points (10 – 1000 ppm) calibration curves standards of 5-*O*-caffeoylquinic acid (CGA), quercetin, kaempferol, apigenin 7-*O*-glucoside and cianidin chloride, with R2 values ranging between 0.9997 and 0.9999. Monocaffeoylquinic acids were expressed as CGA, kaempferol and quercetin derivatives as kaempferol and quercetin equivalents, anthocyanins as cyanidin equivalents. Pure flavonoids, CGA, quercetin and apigenin 7-*O*-glucoside were obtained from Extrasynthèse (Lyon, France)^10^, kaempferol and cyanidin chloride were obtained from Sigma–Aldrich (Saint Louis, MO, United States). Formic acid, acetonitrile and HPLC grade water were purchased from J. T. Baker (Deventer, Holland). All analyses were performed on Arabidopsis and tobacco young leaves and on tobacco petals of fully opened flowers (stage 12, Koltunow et al., 1990). Spectrophotometric analysis of anthocyanins in Arabidopsis seedlings was carried out using the acidified methanol method, as described in Mahmood et al. (2016), except for starting material: 100 mg of frozen seedlings from each genotype (2 weeks after germination) were pooled in a single replicate and homogenized in 500 μl of methanol-HCl (1% v/v). Samples were analyzed spectrophotometrically at 530 and 657 nm wavelengths. Quantification of anthocyanin was calculated by the following equation: A_530_-0.25^∗^ A_657_.

### Statistical Analysis of Data

Statistical significance was tested with Student’s *t*-tests using Sigmaplot 10.1 software or with one-way analysis of variance (ANOVA) with Tukey’s comparison tests, where differences were considered statistically significant at *p* < 0.05.

## Results

### *CcMYB12* Structural Characterization and Phylogenetic Analysis

The artichoke EST database in NCBI was used to retrieve the homologs of *AtMYB12.* The EST with the highest nucleotide sequence similarity, namely GE612454.1 (76% of identity), was used to design a specific primer pair to amplify a 556 bp long cDNA fragment from artichoke leaves. To clone the full length cDNA for *CcMYB12*, 5′ and 3′ RACE analyses were performed and then a forward primer starting from the first codon and a reverse primer containing the stop codon were employed in an end-to-end RT-PCR.

The complete *CcMYB12* coding sequence (GenBank Accession No. MG517449) was 1176 bp in length and predicted to encode a protein of 391 amino-acids (molecular weight: 43.99 kDa theoretical pI: 4.83). A multi-alignment comparison of the deduced protein with MYB TFs annotated in other plant species revealed a high level of sequence identity at the N-terminus, where the characteristic modular structure of the DNA-binding R2R3-MYB domain was identified. In contrast, the rest of the sequence was extremely variable, as for all plant R2R3-MYB transcription factors. The newly isolated artichoke sequence was named *CcMYB12 (Cynara cardunculus MYB12).*

**FIGURE 1:**
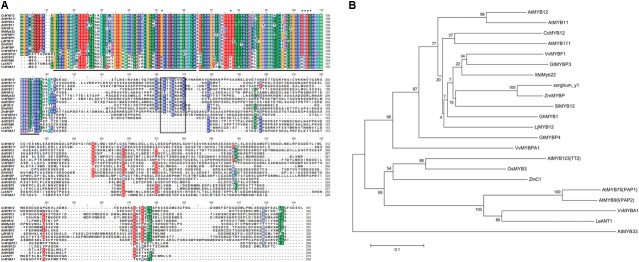
Protein sequence alignment of CcMYB12 deduced amino acid sequence with plant R2R3-MYB transcription factors and phylogenetic analysis. **(A)** Protein sequence alignment of CcMYB12 with representative members of R2R3-MYB-type transcriptional regulators of flavonols, proanthocyanidins and anthocyanins from other plant species. The position of the R2 and R3-type MYB domains is indicated below the alignment by gray bars, the SG7 domain is highlighted by a black box. Key amino acid residues are indicated by asterisks. **(B)** Phylogenetic analysis of deduced amino acid sequences of R2R3-MYB transcription factors present in higher plants. The Neighbor-Joining phylogenetic tree was generated using MEGA software (Tamura et al., 2013). Numerals next to branch nodes indicate bootstrap values from 10000 replications. The bar indicates an evolutionary distance of 0.1%. Protein accession numbers are as follows: apple MdMYB22 (AAZ20438 [*Malus domestica*]); Arabidopsis: MYB11 (NP_191820), MYB12 (CAB09172), MYB111 (AAK97396), AtMYB123/TT2, (CAC40021), AtMYB75/PAP1 (AAG42001), AtMYB90/PAP2 (AAG42002), AtMYB33 (NP_850779); grape: MYBF1 (ACT88298), MYBPA1 (CAJ90831.1) and MYBA1 (BAD18977); gentian: GtMYBP3 (BAM71801) and GtMYBP4 (BAF96934); gerbera: GhMYB1 (CAD87007); lotus LjMYB12 (BAF74782 MYB-related protein [*Lotus japonicus*]); maize: ZmMYBP (P27898) and ZmC1 (AAA33482); rice OsMYB3 (EAY89678 (hypothetical protein [*Oryza sativa*])); sorghum: Y1 (AAX44239); tomato: SlMYB12 (ACB46530) and LeANT1 (AAQ55181). AtMYB33, a GAMYB-like protein, was used as outgroup.

The artichoke CcMYB12 R2 and R3 repeats consist of 53 amino acids (from G^11^to D^64^ residues) and 49 amino-acids (from K^66^ to K^115^), respectively (**Figure 1A**), with the typical helix-turn-helix structure and the highly conserved tryptophan residues implicated in DNA binding [-W-(X19)-W-(X19)-W-(X12)-I-(X18)-W-(X18)-W-] (Stracke et al., 2001). We scanned the CcMYB12 R2R3 amino acid sequence to search key residues probably involved in target promoter specificity. Recently, Heppel et al. (2013) identified key amino acid residues in the MYB R2R3-domain responsible for the specificity of MYB involvement into anthocyanin and proanthocyanidin (PA) biosynthesis. Briefly, the known anthocyanin MYB regulators reported in **Figure 1A** (AtMYB75, AtMYB90, VvMYBA1, and LeANT1) contain an Arg (R) residue at position 52 of the alignment, a Val (V) residue at position 77 and a four amino-acid motif represented by Asp-Asn-Glu-Ile (ANDV) at the position 103–106. In PA and other R2R3 type MYB factors including regulators of flavonol synthesis and CcMYB12, the above mentioned key amino-acid residues are substituted by a Gly (G) at position 52, a Glu (E)/ASP (D) at position 77, and an Asp-Asn-Gln-Val motif (DNEI) at position 103-106, respectively.

Phylogenetic analysis revealed the similarity of CcMYB12 to the flavonol regulators from Arabidopsis, AtMYB111, AtMYB12 and AtMYB11, and other plant MYB proteins such as VvMYBF1, GtMYBP3, MdMyb22, SlMYB12 and ZmMYBP (**Figure 1B**). Similarity was also found to other putative plant flavonol-specific TFs, previously identified (Czemmel et al., 2009), such as *Sorghum bicolor* sorghum_y1, *Lotus japonicus* LjMYB12 and *Gerbera hybrida* GhMYB1 (**Figure 1B**).

The R2R3 domain of CcMYB12 showed the greatest levels of identity (86%) to Arabidopsis AtMYB111 and grape VvMYBF1, to Arabidopsis AtMYB12 and AtMYB11 (84 and 81%, respectively), and to putative gerbera and *Lotus* MYB12 homologs (83 and 79%, respectively). The complete CcMYB12 protein sequence exhibited lower percentages of identities to other proteins, being the C-terminal end very different among MYB TFs. It showed 38% identity to AtMYB111 and 34, 33, 31, 32, and 31% identity to VvMYBF1, LjMYB12, GhMYB1, AtMYB12 and AtMYB11, respectively.

Moreover, CcMYB12 R3 domain did not contain the conserved amino acid signature [DE]Lx2[RK]x3Lx6Lx3R for the interaction with BHLH proteins (Zimmermann et al., 2004), while the SG7 motif (GRTxRSxMK; Stracke et al., 2001) characteristic of flavonol regulators of Arabidopsis was found at the C-terminus of CcMYB12, although partially conserved, given the presence of three amino acid substitutions (GRVSRCVAK).

By a genome walking approach a 3,002-bp-long sequence containing the *CcMYB12* putative promoter region was cloned (GenBank Accession No. MG517450). The search for conserved cis elements in the 1,468 bp upstream the ATG codon using PlantPan and PlantCARE databases revealed the presence of *cis*-regulatory elements putatively involved in the response to hormones (ABA and GA responsive elements), water stress (Myb binding sites MBS, MYBcore), light (ACE elements, GT1-motifs, I-box, HY5 ACGT-containing elements, G-box), biotic stresses (Box-W1) and low temperatures (LTR elements) which are also present in genes of the phenylpropanoid pathway such as *phenylalanine ammonia lyase* (*PAL)*, *chalcone synthase* (*CHS*), *chalcone isomerase* (*CHI*) *dihydroflavonol 4-reductase* (*DFR*), *coumaryl-CoA ligase* (*CL*) in many plant species (Supplementary Table S2; Patzlaff et al., 2003; Hartmann et al., 2005; Prouse and Campbell, 2012).

### Expression Profiles of *CcMYB12* Transcripts in Artichoke

**FIGURE 2:**
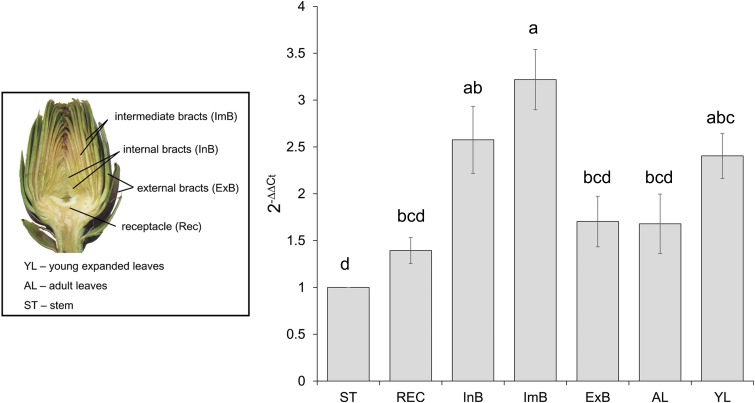
*CcMYB12* gene expression in different organs of artichoke. Quantitative Real Time PCR analysis was performed in the organs depicted in the photograph: stem (St), receptacle (Rec), internal bracts (InB), intermediate bracts (ImB), external bracts (ExB), adult leaves (AL), young leaves (YL), St is the calibrator. Elongation Factor *EF1*α was employed as reference gene. Values are means ± SD of three biological replicates. Bars with different letters are statistically different to each other according to one-way ANOVA Tukey test (*p* < 0.05).

To reveal the expression of *CcMYB12* in artichoke organs, qRT-PCR analysis was performed using total RNA isolated from artichoke heads, leaves and stems. *CcMYB12* transcripts were detected at a higher level in intermediate bracts followed by internal bracts and young leaves; lower levels were detected in external bracts, receptacle, adult leaves and stem (**Figure 2**). To investigate whether *CcMYB12* gene is involved in the regulation of flavonoid production *in vivo*, the expression levels of known artichoke biosynthetic genes, namely, *phenylalanine ammonia lyase* (*PAL1, PAL3*), *p-coumaroyl ester 3′ hydroxylase* (*C3′H*), *hydroxycinnamoyl-CoA shikimate/quinate hydroxycinnamoyl transferase* (*HCT*), *hydroxycinnamoyl-CoA quinate hydroxycinnamoyl transferase* (*HQT*, *HQT1*, *HQT2*), *flavone 3′-hydroxylase* (*F3′H*) and of a putative *flavonol synthase* homolog (*FLS*), were analyzed in all organs (Supplementary Figure S1). In intermediate bracts, where the expression of endogenous *CcMYB12* peaked, the highest levels of *HQT2* and *C3′H* were found, and *PAL1* and *HCT* were highly expressed; in internal bracts *HQT1*, *HQT2* and *C3′H* were among the more expressed genes, while in young leaves *HQT, F3′H* and *FLS* showed the highest levels.

### Recombinant CcMYB12 Protein Interaction With MYBPLANT *Cis*-Motifs

To investigate whether CcMYB12 protein is able to bind *in vitro* to oligonucleotides enclosing MYB-binding cis-motifs, the recombinant CcMYB12 protein was expressed in *E. coli* and its interaction with the MYBPLANT motifs (CCTACC and CCAACC) was investigated by electrophoretic mobility shift assays (EMSA), making use of the ACI and ACII probes (Patzlaff et al., 2003). MYBPLANT motifs were found in the promoters of artichoke structural genes *PAL3a*, *HQT1*, *HQT2*, and *C3′H*, involved in hydroxycinnammic acids and flavonoids synthesis, (De Paolis et al., 2008; Moglia et al., 2009; Sonnante et al., 2010), as well as of several other plant flavonoids biosynthetic genes (Prouse and Campbell, 2012) and *CcMYB12* gene itself.

**FIGURE 3:**
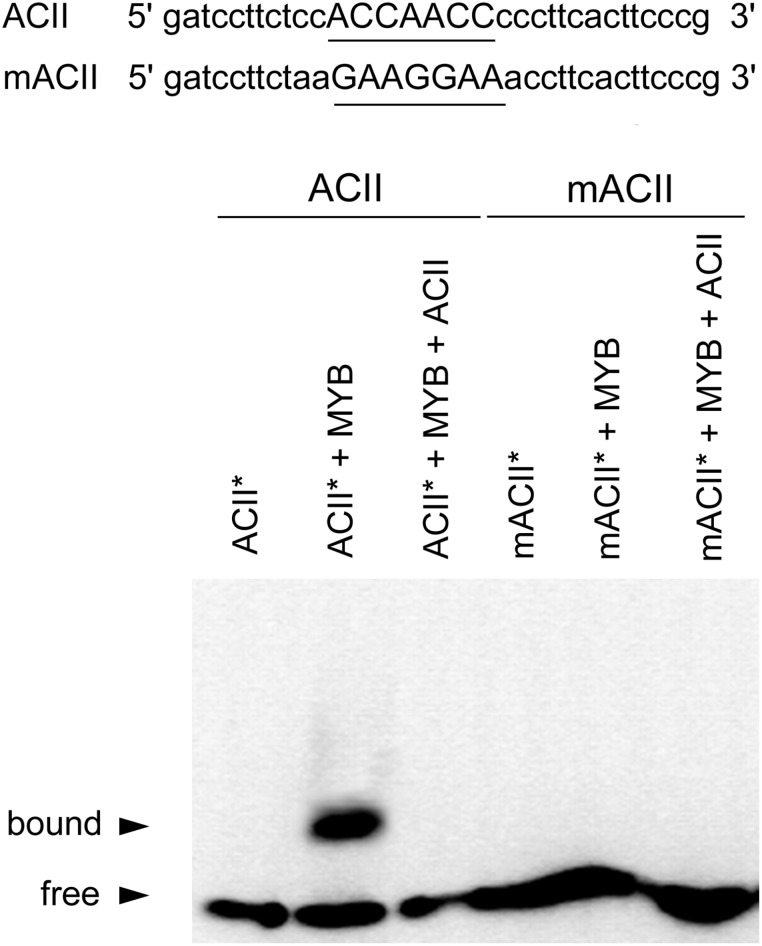
Analyses of CcMYB12 protein binding to AC elements. EMSA output of the purified recombinant CcMYB12 binding to the AC element ACII (left) or to its mutated counterpart (mACII). The biotin-labeled free probe (ACII^∗^) without added protein is designed as control. Binding of recombinant CcMYB12 to biotin-labeled probe ACII^∗^ can be outcompeted by cold competitor ACII. Nucleotide sequences of probes are shown on the top of the figure. The AC or mAC element is underlined.

EMSA analyses revealed that recombinant CcMYB12 was able to bind to ACII probe with high affinity (**Figure 3**), while there was no affinity for ACI probe (Supplementary Figure S2). The CcMYB12 protein did not bind to mutated version of both AC-rich elements, differing by single nucleotide changes, and its binding to biotin-labeled probe ACII^∗^ was outcompeted by cold competitor ACII. Thus, CcMYB12 binds to AC-rich canonical R2R3 MYB-binding site motif MYBPLANT *in vitro*, although in a specifically different manner, preferring ACII to ACI element.

### Overexpression of CcMYB12 in Arabidopsis and Tobacco Plants

In order to gain functional evidence *in vivo*, *CcMYB12* was overexpressed in two different plant systems, Arabidopsis and tobacco. Progenies from twelve independent Arabidopsis primary CcMYB12 transgenic lines were obtained. No morphological changes were observed between WT and empty-vector control plants as well as between transgenic lines, their progenies and WT (data not shown). The expression levels of Arabidopsis genes involved in phenylpropanoid pathway were analyzed by qRT-PCR in leaves of progenies of four CcMYB12 transgenic and WT lines, each sample corresponding to a pool of leaves from four different plants.

**FIGURE 4:**
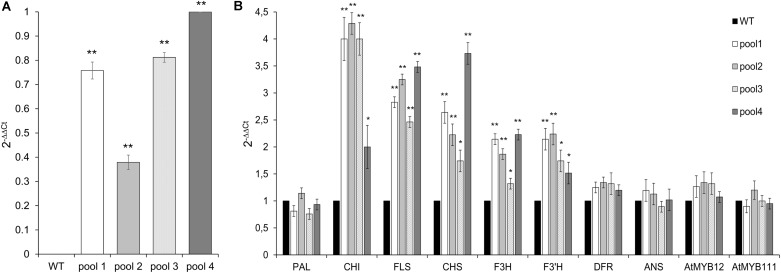
Expression of CcMYB12 transgene **(A)** and of endogenous phenylpropanoid biosynthetic genes **(B)** in leaves of transgenic Arabidopsis plants. qRT-PCR experiments were conducted on cDNAs from various transgenic Arabidopsis T3 plants grouped in 4 different pools (pool 1, 2, 3, and 4), each constituted by 4 plants of the same transgenic T3 line. Actin was employed as reference gene. **(A)** qRT-PCR of *CcMYB12* in transgenic Arabidopsis plants. Pool 4 as calibrator. **(B)** Expression levels of *PAL, CHI, FLS, CHS, F3H, F3′H, DFR, ANS*, *AtMYB12* and *AtMYB111* in CcMYB12 transgenic Arabidopsis plants. WT as calibrator. Values are means ± SD of three biological replicates. Asterisks indicate a statistical difference (^∗^*p*-value < 0.05; ^∗∗^*p*-value < 0.01) between the means for WT and tested transgenic samples, according to Student’s *t*-test.

Although to a different extent, all transgenic lines showed the overexpression of *CcMYB12*, which was absent in WT, as expected (**Figure 4A**). The expression levels of *CHS, CHI* and *FLS* were considerably enhanced in the leaves of transgenic plants compared to WT. *F3′H* and *F3H* levels showed a lower but still significant increase, followed by *DFR* which was only subtly upregulated in transgenic vs. WT plants. Conversely, the expression of *PAL* and *ANS* was unaffected by *CcMYB12.* The expression of endogenous *AtMYB12* and *AtMYB111* were tested in each transgenic line; a modest increase, not exceeding 1.5 fold, was recorded for *AtMYB12* in three out of the four investigated lines (**Figure 4B**), while no effects was recorded for *AtMYB111.*

**FIGURE 5:**
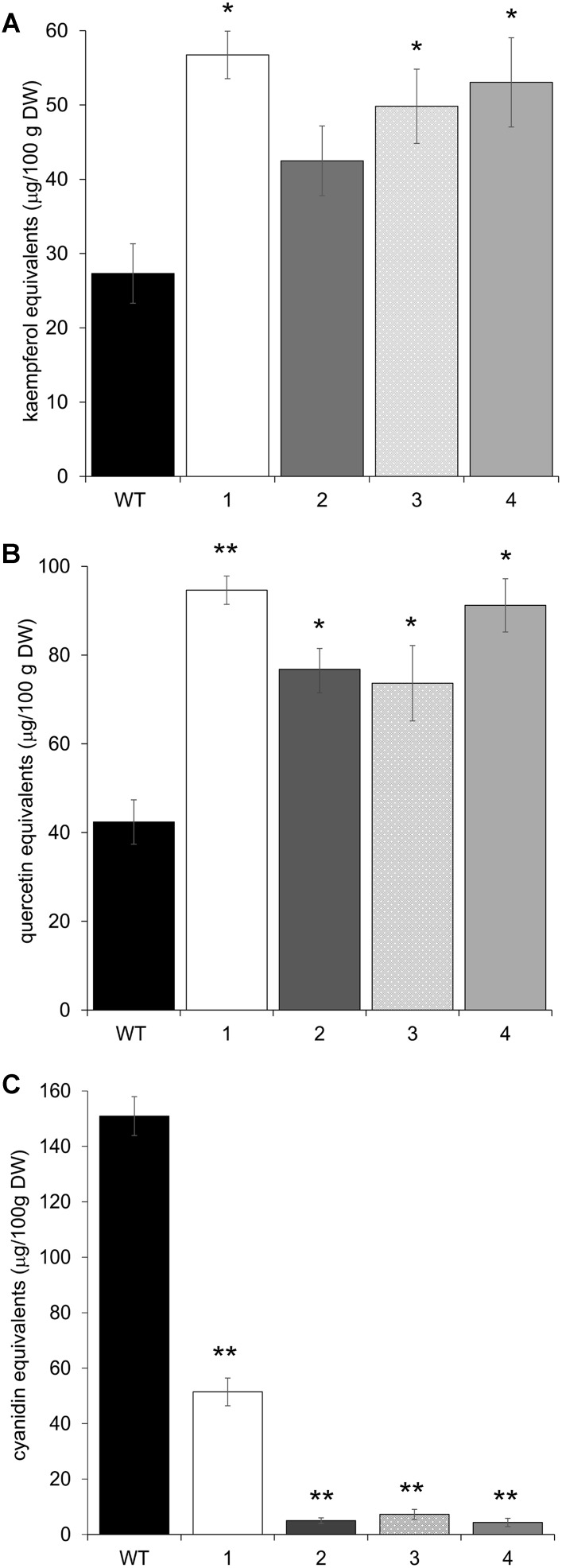
HPLC flavonol and anthocyanin quantification in Arabidopsis plants overexpressing CcMYB12 protein. Leaves were analyzed from wild type (WT) plants and from four different Arabidopsis pools (1–4), each constituted by four plants of the same transgenic T3 line. **(A)** total kaempferols, **(B)** total quercetins, **(C)** total anthocyanins. Error bars indicate the SD of the average of quercetin, kaempferol or cyanidin equivalents determined as triplicates in three independent biological replicates. Asterisks ^∗^ and ^∗∗^ indicate statistically significant differences (*P* < 0.05 and *P* < 0.01 respectively) between the means for WT and tested transgenic samples according to Student’s *t*-test. DW, dry weight.

The upregulation of these early phenylpropanoid genes led to an altered polyphenolic profile in the leaves of transgenic vs. Arabidopsis WT plants. HPLC analyses, performed on methanolic extracts of the same four independent transgenic lines, showed that all plants contained higher amounts of flavonols than WT, in terms of total kaempferols and total quercetins (**Figures 5A,B**). Apigenin content was also investigated, but barely detectable amounts were found in both WT and transgenic plants (data not shown). To check whether these biochemical alterations extend to anthocyanins, the content of these compounds was also determined by HPLC. All transgenic lines showed a significant reduction in anthocyanin levels (**Figure 5C**).

**FIGURE 6:**
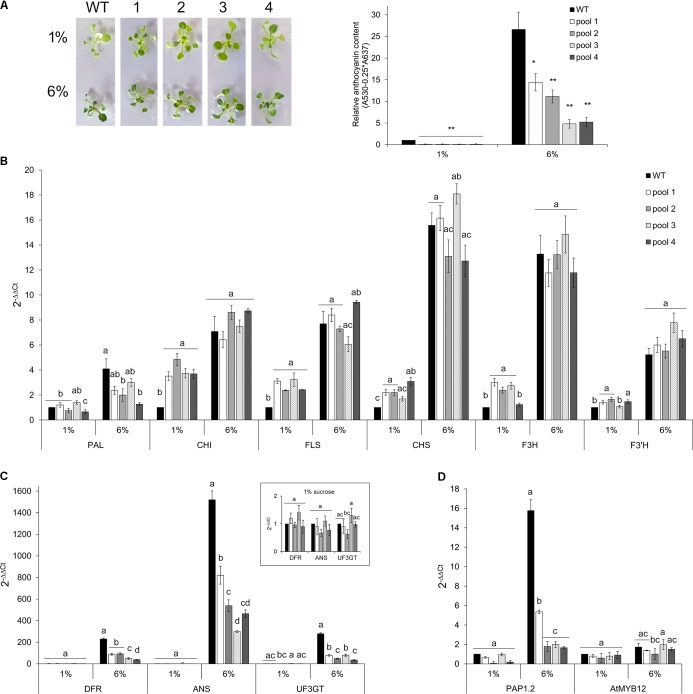
Anthocyanin content and qRT-PCR analysis in Arabidopsis seedlings grown on control and anthocyanin inductive medium. **(A)** Left panel: 20-days-old Arabidopsis wild type (WT) and CcMYB12 seedlings (1, 2, 3, and 4) grown in MS medium supplemented with 1 or 6% sucrose under long day condition. Right panel: Anthocyanin content in 2-weeks-old WT and CcMYB12 seedlings grown in 1 and 6% sucrose. Values are reported as relative to WT 1% sucrose, set as 1. Data represent mean values (+SD, *n* = 3). Within each growth condition, asterisks ^∗^ and ^∗∗^ indicate statistically significant differences (*P* < 0.05 and *P* < 0.01 respectively), between the means for WT and tested transgenic samples according to Student’s *t*-test. **(B–D)** qRT-PCR analysis of early **(B)** and late **(C)** flavonoid biosynthetic genes, and AtPAP1 and AtMYB12 TFs **(D**) in seedlings grown in 1 and 6% sucrose. Upper panel in **(C)** represents late biosynthetic genes with a low expression level in 1% sucrose condition, with the appropriate axis scale. Actin was employed as reference gene. WT grown in 1% sucrose is the calibrator. Values are means ± SD of three biological replicates. Bars with different letters are statistically different to each other according to one-way ANOVA Tukey test (*p* < 0.05).

To further investigate whether and to what extent *CcMYB12* is a negative regulator of anthocyanin biosynthesis in Arabidopsis, WT and CcMYB12 overexpressing lines were grown either on control (1%) or on anthocyanin inductive medium (6% sucrose) (Teng et al., 2005; Solfanelli et al., 2006; Rubin et al., 2009). Under inductive condition anthocyanin production increased, in both WT and transgenic seedlings with respect to the control, but the levels of these metabolites remained significantly lower in CcMYB12 than in WT seedlings (**Figure 6A**). On anthocyanin inductive medium all the flavonoid early (EBGs) and late (LBGs) biosynthetic genes tested were up-regulated in WT and CcMYB12 seedlings. Nevertheless, while in control condition, *CHI, FLS, CHS, F3H, F3′H* transcript levels were significantly higher in CcMYB12 than in WT, on 6% sucrose the transcript levels of these genes were not significantly different between WT and CcMYB12 lines (**Figure 6B**). Interestingly, LBGs showed comparable transcript levels between WT and CcMYB12 transgenic seedlings on 1% sucrose (panel in **Figure 6C**), whereas they were significant downregulated in the latter samples on high sucrose condition (**Figure 6C**). The anthocyanin regulatory gene *PAP1* showed an expression pattern similar to that of LBGs, whereas the endogenous flavonol regulator AtMYB12 was unaffected by the transgene (**Figure 6D**).

**FIGURE 7:**
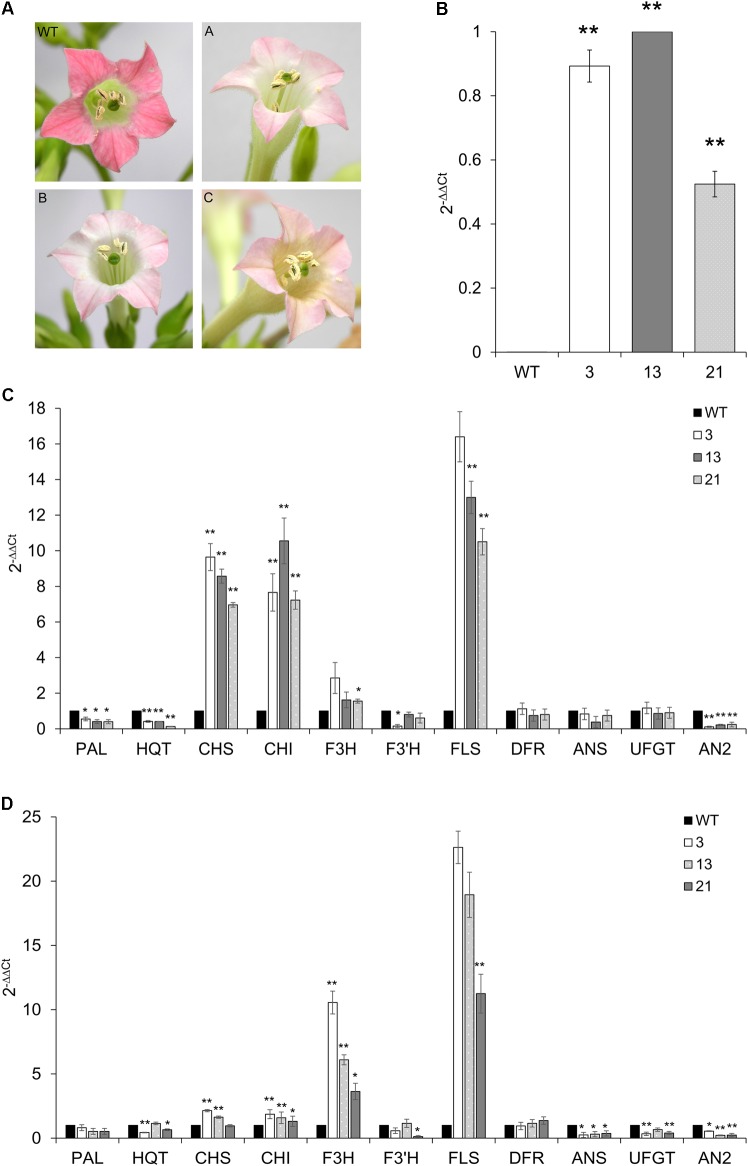
Expression of *CcMYB12* transgene and of endogenous phenylpropanoid biosynthetic genes in CcMYB12-expressing tobacco plants. **(A)** Color of tobacco flowers in transgenic CcMYB12 lines. The pigmentation of corolla limbs of three different transgenic lines expressing CcMYB12 was compared with corolla from WT. **(A)** line 3, **(B)** line 21, and **(C)** line 13. **(B)** qRT-PCR of exogenous *CcMYB12* in transgenic tobacco plants. Line 13 as calibrator. **(C,D),** Expression levels of the early biosynthetic genes, namely *PAL, HQT, CHS, CHI, F3H, F3′H, FLS*, and *ANS*, *DFR*, *UFGT* and *AN2in* transgenic tobacco leaves **(C)** and flowers **(D)**. WT as calibrator. qRT-PCR experiments were conducted on cDNAs from various transgenic tobacco T3 plants. Three independent preparations of total RNA from each line were assayed in triplicate. Actin was employed as reference gene. Values are means ± SD of three biological replicates. Asterisks indicate a statistical difference (^∗^*p*-value < 0.05; ^∗∗^*p*-value < 0.01) between the means for WT and tested transgenic samples, according to Student’s *t*-test.

Tobacco primary transformants and their progenies showed clear pigmentation alterations in fully opened flowers: the floral limbs of CcMYB12 plants were markedly less colored compared to WT plants or tobacco plants carrying the GUS reporter gene, indicating reduced levels of anthocyanins (**Figure 7A**).

Quantifications of transcripts of phenylpropanoid genes and of flavonoids were performed in WT and T3 tobacco leaves and petals from flowers at stage 12. Three independent T3 transgenic lines were assayed, and all of them showed the expression of *CcMYB12*, with plants 3 and 13 displaying the highest levels (**Figure 7B**). As in transgenic Arabidopsis, the expression of *CHS, CHI, FLS* and, although less markedly, of *F3H*, increased in tobacco transgenic leaves compared to WT, whereas *DFR* and *ANS (anthocyanin synthase)* genes were not affected. *PAL*, *HQT* and *AN2* (a R2R3-MYB TF that regulates anthocyanin accumulation in tobacco) were significantly downregulated, and *F3′H* was significantly downregulated in plant 3 only (**Figure 7C**). In tobacco transgenic flowers, high expression of *F3H* and *FLS* were found, *CHS* and *CHI* transcripts slightly, but still significantly, increased, while no differences were found in the expression levels of *PAL* and *DFR*, with respect to WT (**Figure 7D**). Furthermore, the expression of *CcMYB12* caused a significant downregulation of *ANS, UFGT* (*glucose: flavonoid 3-O-glucosyltransferase*) and *AN2* in the flowers of all the transgenic plants assayed, whereas *HQT* and *F3′H* did not show a trend fully matching with the expression of the transgene (**Figure 7D**).

**FIGURE 8:**
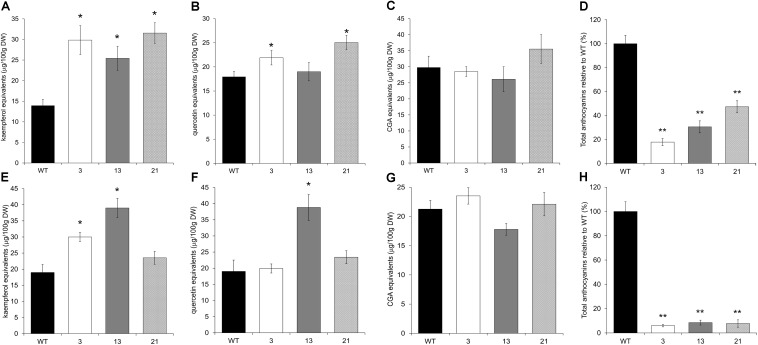
HPLC analyses of flavonoids and caffeoyl-quinic acids in leaves and flowers of tobacco plants overexpressing CcMYB12 protein. Leaves **(A–D)** and flowers **(E–H)** from WT and T3 transgenic tobacco lines 3, 13, and 21 were tested. Total kaempferols **(A,E)**, total quercetins **(B,F)**, chlorogenic acid (CGA; **C)**, total caffeoyl-quinic acids (CQAs; **G),** total anthocyanins **(D,H)**. Total anthocyanin content is relative (in percentage) to the mean levels of the WT line. Error bars indicate the SD of the average of kaempferol, quercetin, CGA or cyanidin equivalents, and CGA determined as triplicates in three independent biological replicates. Asterisks indicate a statistical difference (^∗^*p*-value < 0.05; ^∗∗^*p*-value < 0.01) between the means for WT and for tested transgenic samples, according to Student’s *t*-test. DW, dry weight.

HPLC analyses of flavonoids in tobacco leaves showed a significant increase of kaempferol in the transgenic lines, and of quercetin in two of the three tested lines (**Figures 8A,B** and Supplementary Figure S4). The apigenin 7-*O*-glucoside content was also increased in all transgenic lines, although significantly only in plant 3 (Supplementary Figure S3), whereas the CQA content, in particular CGA, was similar to WT (**Figure 8C** and Supplementary Figure S4).

Fully developed flowers of CcMYB12 transgenic tobacco lines showed a high content of flavonols as kaempferol derivatives when compared to WT flowers, whereas a clear trend did not emerge for quercetin derivatives (**Figures 8E,F**). Total CQAs were not affected (**Figure 8G**), while apigenin 7-*O*-glucoside was not detected in both WT and transgenic flowers.

HPLC analysis revealed that the depigmentation exhibited by *CcMYB12* transgenic flowers was due to a severe reduction, up to 90%, of anthocyanin levels if compared to WT flowers (**Figure 8H** and Supplementary Figure S4). A strong reduction of anthocyanins, spanning from 50 to 80%, was also observed in the leaves of transgenic plants (**Figure 8D**).

Therefore, the results obtained from qRT-PCR and HPLC analyses suggest that, while promoting the accumulation of flavonols, mainly kaempeferol derivatives, *CcMYB12* interferes with anthocyanin accumulation in both Arabidopsis and tobacco heterologous models.

## Discussion

Direct consumption of artichoke heads represents an important source of polyphenols, inulin and flavonoids, healthy compounds with antioxidant and therapeutic activity. Artichoke leaves are also rich in these metabolites. The profiling of nutraceuticals, their qualitative - quantitative variation according to environmental conditions on artichoke landraces, genotypes and plant organs are issues which have been approached only recently (Lombardo et al., 2010; Negro et al., 2012; de Falco et al., 2016). A fine metabolic characterization is the prerequisite for programs of germplasm preservation, selection and propagation of this healthy Mediterranean crop.

Regulation of phenylpropanoid accumulation is achieved at the transcription level, and R2R3-MYB proteins act as flavonoid transcriptional regulators (Liu et al., 2015). The first R2R3-MYB controlling flavonol biosynthesis, *MYB12*, has been characterized in Arabidopsis, where *AtMYB12* mainly controls flavonol production, acting as a regulator on *CHS, CHI, F3H* and *FLS* (Mehrtens et al., 2005; Stracke et al., 2007). *AtMYB12* shows a high degree of functional similarity to *AtMYB11* and *AtMYB111*, with differential spatial activities (Stracke et al., 2007). Interestingly, *AtMYB12* overexpression in other plant species emphasized the complex regulation of plant secondary metabolism. If *AtMYB12* overexpression in tomato leads to flavonol and CGA accumulation (Luo et al., 2008), in kale it triggers over accumulation of total phenolics and flavonols (Lännenpää, 2014), while in tobacco plants and cell cultures its influence is restricted to flavonol and rutin accumulation (Luo et al., 2008; Misra et al., 2010). Moreover, characterization of endogenous MYB12 TF in plants as grape (*VvMYBF1*, Czemmel et al., 2009; Czemmel et al., 2017), tomato (*SlMYB12*, Ballester et al., 2010) and gentian (*GtMYBP3* and *GtMYBP4*, Nakatsuka et al., 2012) showed how flavonoid accumulation, their compositional diversity and targeted biosynthetic genes could be differentially regulated across plant species. Hence, in order to improve flavonoid content and composition, there is an effective need to examine regulatory mechanisms of flavonoid biosynthesis, species by species. In face of its relevance for the Mediterranean diet, only genes related to chlorogenic acid (CGA) biosynthesis and early phenylpropanoid biosynthetic pathways have been investigated in artichoke thus far (Comino et al., 2007, 2009; De Paolis et al., 2008; Moglia et al., 2009; Sonnante et al., 2010; De Palma et al., 2014). Hence, in this study we aimed at identifying and characterizing *MYB12* from artichoke, as one of the main regulators of flavonol biosynthesis.

Plant MYB factors can be categorized into subgroups, based on Arabidopsis conserved amino acid motifs previously detected in the C-terminus, outside the MYB domain (Stracke et al., 2001). These subgroups may cluster factors that share the same function. Our phylogenetic analysis reveals that *CcMYB12* joins the clade of putative flavonol MYB factors from different plant species. It shows high level of similarity to the Arabidopsis flavonol regulator *AtMYB111*, to the functionally redundant *AtMYB12* and *AtMYB11*, and to grape V*vMYBF1*, gerbera *GhMYB1*, gentian *GtMYBP3*, tomato *SlMYB12* and maize *ZmP1*.

A detailed analysis of protein conserved domains shows that CcMYB12 owns key amino acid residues, within the canonical R2R3 repeat domains, probably involved in target promoter specificity for flavonol biosynthesis (i.e., Gly^52^, E^77^, and DNEI^103-106^), also conserved in AtMYB12 and its homologs. CcMYB12 lacks the bHLH binding site for interaction with bHLH proteins (Zimmermann et al., 2004), as other plant flavonol specific MYB TF, thus showing cofactor independency. Although the sequence similarity between R2R3-MYB proteins is generally confined to the N-terminus, the CcMYB12 presents a motif (GRVSRCVAK) outside the R2R3 domains that shares 67% identity with the SG7 motif (GRTxRSxMK). This motif typifies R2R3-MYB proteins that regulate flavonol biosynthesis (Stracke et al., 2001). These evidences are in support of the hypothesis that *CcMYB12* is indeed a MYB TF involved in regulation of flavonol biosynthesis. This hypothesis is also corroborated by transcriptional data, as they reveal that *CcMYB12* is highly expressed in artichoke intermediate and internal bracts, and in young leaves. The intermediate bracts, where *CcMYB12* is more expressed, show high levels of the known biosynthetic flavonoid genes *HQT1*, *HQT2*, *C3′H*, *HCT* and to a lesser extent of *PAL3a*. Although the correlation of *CcMYB12* expression with biosynthetic flavonoid gene levels and flavonoid accumulation in artichoke tissues cannot be established directly, as not all structural genes have been isolated and characterized yet, transcriptional and metabolic data seem to overlap quite nicely. We note in fact that artichoke heads are very rich in caffeoylquinic acids and in some apigenin derivatives (apigenin, luteolin, naringenin and their related glycosides), mainly in receptacles and inner bracts (Fratianni et al., 2007; Negro et al., 2012). Anthocyanins (specifically cyanidin 3-*O*-β-glucoside) are present in the water-soluble fractions of artichoke head extracts (de Falco et al., 2016), whereas flavonoids are more concentrated in leaves (Lattanzio et al., 2009; Negro et al., 2012; de Falco et al., 2016). Accordingly, *CcMYB12* is expressed both in flavonoid rich leaves and in capitula tissues, the latter characterized by high levels of caffeoylquinic acids and apigenin derivatives.

The maize P protein (Grotewold et al., 1994) and more recently AtMYB11, AtMYB12 and AtMYB111 (Stracke et al., 2007) were shown to bind to ACC(A/T)ACC(A/C/T) motifs, commonly referred to as MYBPLANT, P-box, MRE or AC-elements (Patzlaff et al., 2003; Prouse and Campbell, 2012). Such elements are present in the promoter regions of artichoke phenylpropanoid genes such as *HQT1*, *HQT2*, *PAL3a* and *C3′H* (De Paolis et al., 2008; Sonnante et al., 2010) as well as in the promoter of *CcMYB12* itself, as reported here. We show that the recombinant protein CcMYB12 specifically recognizes the ACII probe containing the MYBPLANT motif “ACCAACC”, whereas it does not bind to the other probe tested (ACI: ACCTACC). Although, data on the interaction between MYBs and their target DNAs could differ moving from *in vitro* to *in vivo* analyses (Prouse and Campbell, 2012), we interpret the results of our *in vitro* mobility shift assay to mean that CcMYB12 is indeed a TF and that MYB12 TFs from different plant species retain a different specificity toward target DNA motifs. Thus, the transcriptional profiles of early biosynthetic genes and *in vitro* EMSA convey to suggest a role of *CcMYB12* in controlling flavonol biosynthesis in artichoke.

Being artichoke recalcitrant to genetic transformation, direct evidence on the role of *CcMYB12* cannot be gained. To overcome this problem, we ectopically expressed *CcMYB12* into the model systems Arabidopsis and tobacco, and performed targeted transcriptional and metabolic analyses on Arabidopsis and tobacco leaves and tobacco flowers. Differently from Arabidopsis, tobacco produces CGA (Tamagnone et al., 1998; Niggeweg et al., 2004) and its flowers accumulate the flavonols quercetin and kaempferol in the form of various glycosides (Luo et al., 2007; Passeri et al., 2017) as well as anthocyanin derivatives, being cyanidin 3-*O*-rutinoside the dominant anthocyanin (Aharoni et al., 2001; Luo et al., 2007; Nakatsuka et al., 2007; Passeri et al., 2017).

The main effects shared by Arabidopsis leaves, tobacco leaves and flowers as a result of *CcMYB12* ectopic expression consist in the metabolic flux diversion from anthocyanins to flavonols, which is conceivably mainly due to the upregulation of *FLS*. With a few exceptions (see below), these effects resemble those reported in the same model systems as a consequence of the ectopic expression of *AtMYB12*, to suggest that *CcMYB12* is a putative homologue of *AtMYB12.*

The expression of *CcMYB12* induces the upregulation of the early biosynthetic genes *CHS, CHI, F3H* and *FLS* both in Arabidopsis and tobacco leaves. The co-regulation of multiple genes of the flavonoid pathway was previously reported as a result of the overexpression of *AtMYB12* in different species such as Arabidopsis, tobacco and tomato (Hartmann et al., 2005; Stracke et al., 2007; Luo et al., 2008; Misra et al., 2010; Pandey et al., 2015). *CHS, CHI, F3H* and *FLS* genes were fully characterized in Arabidopsis; they carry identical *cis*-elements in their promoters which are required for flavonol biosynthesis, including the MYBPLANT motif, also recognized by the newly identified artichoke MYB TF. Differently from what observed in Arabidopsis and tobacco leaves, *CcMYB12* overexpression does not induce *CHS* expression in the flowers of all transgenic plants.

As stated above, few differences characterize *CcMYB12* with respect to *AtMYB12* function, both in target gene regulation and metabolite profiles of transgenic plants. Firstly, a previous study showed that both *AtMYB12* Arabidopsis gain- and loss-of-function mutants do not display seedlings with altered content of anthocyanins with respect to WT in culture medium with no sucrose (Mehrtens et al., 2005), whereas here we show that the levels of these metabolites decrease in *CcMYB12* transgenic Arabidopsis leaves as well as in transgenic seedlings, regardless of the sucrose concentration. Moreover, *AtMYB12* does not appear to induce *F3′H* gene expression strongly (Mehrtens et al., 2005; Luo et al., 2008), while *CcMYB12* does on leaves and, although not in all transgenic lines, on seedlings too. In tobacco, the inverse correlation between anthocyanins and flavonols in AtMYB12 transgenic flowers was previously interpreted as the result of the competition between DFR and FLS for dihydroflavonol precursors (Luo et al., 2008). Likewise, we argue that the flux diversion between flavonols and anthocyanins in *CcMYB12* transgenic tobacco leaves and flowers is also accountable to the competition between FLS and DFR, being *FLS* gene upregulated and *DFR* unaffected in these organs by the transgene. Such a competition occurs particularly in floral limbs where CcMYB12 expression leads to a severe reduction in *ANS* steady state levels. Despite this parallelism, *AtMYB12* and *CcMYB12* functions also diverge in tobacco: while *AtMYB12* increases the levels of CQA, the levels of these metabolites are unaffected both in CcMYB12 transgenic leaves and flowers. In the absence of a clear effect of *AtMYB12* on genes encoding *HQT*s and *C3′H*, the CQA increment in AtMYB12 transgenic tobacco flowers was previously accounted to the induction of *PAL* (Luo et al., 2008). Thus, embracing the assumption that *PAL* activity limits flux through phenylpropanoid metabolism in tobacco (Howles et al., 1996), in accordance with Luo and colleagues we reason that the lack of CQA increment in CcMYB12 transgenic tobacco is due to the inability of this TF in promoting the upregulation of *PAL* as well as of *HQT.* Better still, we note that the levels of CGA in transgenic tobacco leaves remain unaltered with respect to the WT, despite the fact that both *PAL* and *HQT* are downregulated by *CcMYB12*.

As stated above, in Arabidopsis leaves *CcMYB12* not only clearly increases the steady state levels of *CHS*, *CHI* and *FLS*, but also that of *F3′H*, which is necessary for the production of quercetin. In turn, both kaempferol and quercetin derivatives are concomitantly increased in transgenic Arabidopsis plants. Conversely, in transgenic tobacco plants we observe a more variegated patter concerning these flavonols, since both in leaves and flowers kaempferol derivatives are increased, whereas the increment of quercetin derivatives is less marked and it does not occur in the leaves and flowers of all CcMYB12 transgenic lines. This is consistent with the qRT-PCR data showing that in tobacco *F3′H* is not among the CcMYB12 primary target genes. Incidentally, we note that a different and organ-specific regulatory mechanism between the kaempferol and quercetin branches has been highlighted in tobacco flowers as a consequence of the ectopic expression of the proanthocyanidin regulator MYBPA1 from grape (Passeri et al., 2017). Also the different effect exerted in tomato on the production of kaempferol and quercetin type flavonol by the *MYB*s genes *C_1_* or *Lc* vs. *AtMYB12*, was attributed to the different ability of these TFs in inducing the endogenous *F3′H* gene (Bovy et al., 2002; Luo et al., 2008).

In tobacco, in addition to the flux diversion from anthocyanins to flavonols, *CcMYB12* triggers changes in the levels of the flavone apigenin. This is the major flavone present in tobacco leaves and in developing tobacco flowers (Nakatsuka et al., 2006; De Palma et al., 2014). In keeping with this observation, we did not detect apigenin in fully developed flowers (stage 12), however its content increased in the *CcMYB12* transgenic leaves if compared to WT, likely as a result of the marked upregulation of *CHS* and *CHI* in these organs. Moreover, it negatively regulates *UFGT* and *AN2*.

Finally, we note that in Arabidopsis seedlings the decrease in anthocyanins in CcMYB12 overexpressing lines might be accountable to two different, although not mutually exclusive, mechanisms depending on growing conditions: under control condition CcMYB12 promotes *FLS* upregulation and consequently the flux diversion from anthocyanins to flavonols. Conversely, under anthocyanin inductive condition, *CcMYB12* seems to directly interfere with the steady state levels of *PAP1*, one of the main regulators of the biosynthesis of these pigments and, in turn, it negatively regulates key LBGs such as *DFR*, *ANS* and *UFGT*. Further analyses will be needed to prove this hypothesis and disentangle the mechanism by which CcMYB12 represses *PAP1*.

In sum, through the isolation of *CcMYB12* and its expression in heterologous models, here we shed light on a regulator of flavonol biosynthesis from the globe artichoke, producing an accumulation of flavonols and a reduction of anthocyanins. The differences between host plants (Arabidopsis and tobacco), plant organs (tobacco leaves vs. flowers) and plant growing conditions (anthocyanin inductive vs. control condition) in the sets of the genes targeted by *CcMYB12* suggest a different, species- and organ- specific and environmental dependent – capacity of this TF to interact with endogenous genes of the flavonoid pathway. Likewise, in artichoke the interaction of *CcMYB12* with other endogenous genes as well as its expression profiles might vary according to the organs and in response to different stimuli and/or developmental stages.

Further analyses on *CcMYB12* expression profiles and its interaction with flavonoid regulatory and biosynthetic genes in different organs and under different plant growing conditions will advance our understanding on genetic and environmental determinants underlying flavonol biosynthesis in artichoke. This is the prerequisite to develop agronomic and biotechnological strategies aimed at selecting and breeding artichoke germplasm with enhanced levels of healthy compounds.

## Author Contributions

EB and GS conceived and designed the research. EB, WS, DD, DN, and VP performed the experiments. EB, WS, DD, DN, ADL, FP, and GS analyzed and interpreted the data. EB, FP, and GS contributed to reagents, materials, and analysis tools. EB, FP, and GS wrote and revised the paper.

## Conflict of Interest Statement

The authors declare that the research was conducted in the absence of any commercial or financial relationships that could be construed as a potential conflict of interest.

## Footnotes

1 http://www.ncbi.nlm.nih.gov/

2 https://blast.ncbi.nlm.nih.gov/Blast.cgi

3 http://www.ebi.ac.uk/Tools/msa/clustalo/

4 http://www.mbio.ncsu.edu/bioedit/bioedit.html

5 http://www.megasoftware.net/

6 http://web.expasy.org/protparam/

7 https://www.ebi.ac.uk/interpro/

8 http://PlantPAN.mbc.nctu.edu.tw

9 http://bioinformatics.psb.ugent.be/webtools/plantcare/html/

10 http://www.extrasynthese.com

## Abbreviations

CGA: chlorogenic acid
CQAs: caffeoyl-quinic acids
TF: transcription factor
