# Supplementary material for: Isolation and Characterization of the Flavonol Regulator CcMYB12 From the Globe Artichoke [Cynara cardunculus var. scolymus (L.) Fiori]
Source: Front Plant Sci. 2018 Jul 4;9:941. doi: 10.3389/fpls.2018.00941 (PMC6042477; doi:10.3389/fpls.2018.00941)
Supplement: Supplementary file 1 [file Table_1.DOCX]

**Supplementary Table S1.** List of primers used in this research.

| **Gene**  **(Acc.no./gene name)** | **Primer name** | **Sequence 5′-3′** | **Purpose** |
| --- | --- | --- | --- |
| *CcMYB12* | MYB12F1 | TATAACAAGCGAAGAAGAAGAAAT | cDNA isolation and cloning |
|  | MYB12R1 | CAATAACTCGGAAGAACTACTC |  |
|  | 5RACE_MYB_3 | TCATCTGGTCCTAGATCTTGATTTTCTCTCTC |  |
|  | 5RACE_MYB_2 | AGAACTTGCTGATATCCATTAATTCATCTACTTG |  |
|  | 5RACE_MYB_1 | CAATAACCCATCTAAA |  |
|  | 3RACE_MYB_F1 | GCGGGGAGAGCATGATCAATACA |  |
|  | 3RACE_MYB_F2 | GACGCAAGATGTACAAATATTTTAGAGGGA |  |
|  | 3RACE_MYB_F3 | AATAACAAGAAACCACCACCATCTGCTA |  |
|  | CcMyb12-FWatg | ATGGGAAGAACTCCTTGCTGTC |  |
|  | CcMyb12-RVstop | TTACAAAACTGAACCAGAAAACCCTA |  |
|  | pE-MYB12-R | CAAAACTGAACCAGAAAACCCTAGCA |  |
|  | GwCcMYB12_GSP2 | TAACCCTTCTTTCTGACAGCAAGGAGTTCT | Promoter isolation |
|  | GwCcMYB12_GSP1 | AGACCTCCAAGAACCTTCTCCATTGGCTTGA |  |
|  | GW-MYB12-F | CACCATGGGAAGAACTCCTTGCT | Transgenic plants generation and selection |
|  | MYB1_F | AATTCTTGAGCTTGGATTCATCAGAA |  |
| *nptII* | nptII-fw | GCTCAGAAGAACTCGTCAAGAAG | Transgenic plants selection |
|  | nptII-rv | TGGAGAGGCTATTCGGCTATGA |  |
| *hptII* | hptII-F2 | CCTAGAATGAAAAAGCCTGAACTC |  |
|  | hptII-R2 | CAGTCAATGACCGCTGTTAT |  |
| *CcMYB12* | MYB12RTfw1 | TCATCAGAAGCTATGGGGTTTTG | qRT-PCR |
|  | MYB12RTrv1 | GTTGTTCCTGATCATCTTCTTCC |  |
| ABK79689 | HQTcomSNP-F | ACGGCTGGGTAACGATGG | qRT-PCR on artichoke |
|  | HQTcomSNP-R | CAATGCTACAATCCGCTTCA |  |
| AM690438 | RT-HQT-F2 | TTTGACCACATCGAGTACCACGCT |  |
|  | RT-HQT-R2 | TCGTTCTTGGCTGAGGCTTTGAGA |  |
| EU839580 | RT_HQT2_F | CACCACACTGACCAAAATGG |  |
|  | RT_HQT2_R | TATACCGGTAGTCGGGTCCA |  |
| AAZ80046 | HCTgap-F | ACCGAATCAACACCAACCAT |  |
|  | HCTgap-R | GCTTGATTGACTTTGAAGAAACG |  |
| AM418560 | MB_Pal1F | TGTAGATCGTACCCGTTGTATAGG |  |
|  | MB_Pal1R | CGTGAACACCTTATCGAACTCTTC |  |
| AM418586 | MB_Pal3F | GGAGTTTGATAAGGTGTTTACAGC |  |
|  | MB_Pal3R | GCAGATTCTAGGAGATTGGAAGAG |  |
| FJ225121 | cynRT C3'H F | GGATTTGATTTGGGCGGATTATGGC |  |
|  | cynRT C3'H R | TCTTCTCTGATGGGTCGAAGGTGC |  |
| HM153534 | RTCYN F3'H F | CACTCTCCTTGCCACGGATTGC |  |
|  | RTCYN F3'H R | GACTGGGTCGGAATTCAAGCGG |  |
| GE604603.1 | CcFLS_FW2 | GGAAGCCATAGCCAAACCTGC |  |
|  | CcFLS_RV2 | TGGCCAAACCCTATGAAACAAGT |  |
| EU442190 | El_RT_F | TCCCAGGCTGATTGTGCTGTCCTTAT |  |
|  | El_RT_R | ATGCTCACGGGTCTGACCATCCTTA |  |
| *At2g37040* | AtPAL1fw1 | ATCGAAGTGATCCGTTACGC | qRT-PCR on transgenic Arabidopsis plants |
|  | AtPAL1rv1 | ACTCCGATTGGTGTTCCTTG |  |
| *At5g13930* | AtCHSfw1 | TGAGAACCATGTGCTTCAGG |  |
|  | AtCHSrv1 | CAGATGCATGTGACGTTTCC |  |
| *At3g55120* | AtCHIfw | TTTGTACCGTCCGTCAAGTC |  |
|  | AtCHIrv | CAATGACGGTGAAGATCACG |  |
| *At3g51240* | AtF3Hfw | TCAGATCGTTGAGGCTTGTG |  |
|  | AtF3Hrv | ATGTCGAAACGGAGCTTGTC |  |
| *At5g42800* | AtDFRfw | GTCGGTCCATTCATCACAAC |  |
|  | AtDFRrv | TGAGCGTTGCATAAGTCGTC |  |
| *At5g08640* | AtFLSfw1 | CCGTCGTCGATCTAAGCGAT |  |
|  | AtFLSrv1 | CGTCGGAATCCCGTGGT |  |
| *At5g07990* | AtF3PHfw1 | GCTCTCGCCGGAGTATTCAA |  |
|  | AtF3PHrv1 | CCAGCGACGCCTTGTAAATC |  |
| *At4g22880* | AtANS-Fw | AGAGAAGAGAGATCTATCAATTTGGCCT |  |
|  | AtANS-Rv | GCGTACTCACTCGTTGCTTCTATG |  |
| *At5g49330* | AtMYB111-fw | CAATGTTTCTCACAACCTAAGGAGC |  |
|  | AtMYB111-rv | CCAAAGACTCTCCTTCAAAATTACCA |  |
| *At2g47460* | AtMYB12FW | AACCAAGGGAATCTCGACTGTCT |  |
|  | AtMYB12RV | CCCAATCGATAAACTCATCCGT |  |
| *At1g56650* | AtPAP1-FW2 | TGCTGGAAGATTACCTGGTCG |  |
|  | AtPAP1-RV2 | AGTGCCGGTGTTGTAGGAATG |  |
| *AT5G54060* | AtUF3GT-FW1 | GTGTTCTGCGCTTTCGGTAG |  |
|  | AtUF3GT-RV3 | AGGAGGCTTAATGGCAACC |  |
| *At3g18780* | AtACT2-Fw | ACCCGATGGGCAAGTCATC |  |
|  | AtACT2-Rv | CGAGGGCTGGAACAAGACTTC |  |
| X78269 | NtPAL_RT_F | ATTGAGGTCATCCGTTCTGC | qRT-PCR on transgenic tobacco plants |
|  | NtPAL_RT_R | ACCGTGTAACGCCTTGTTTC |  |
| AF311783 | NtCHS_RT_F | TTGTTCGAGCTTGTCTCTGC |  |
|  | NtCHS_RT_R | AGCCCAGGAACATCTTTGAG |  |
| AB213651 | NtCHI_RT_F | GTCAGGCCATTGAAAAGCTC |  |
|  | NtCHI_RT_R | CTAATCGTCAATGCCCCAAC |  |
| AB289450 | NtF3H_RT_F | CAAGGCATGTGTGGATATGG |  |
|  | NtF3H_RT_R | TGTGTCGTTTCAGTCCAAGG |  |
| EF421429 | NtDFR_RT_F | AACCAACAGTCAGGGGAATG |  |
|  | NtDFR_RT_R | TTGGACATCGACAGTTCCAG |  |
| AB289447 | NtANS_RT_F | TGGCGTTGAAGCTCATACTG |  |
|  | NtANS_RT_F | GGAATTAGGCACACACTTTGC |  |
| AB289449.1 | NtF3'H_RT_F | AGAATTGCATCCGAGAGTTGTGAG |  |
|  | NtF3'H_RT_R | AGAAATCTTTCGGGCCTAAACTCC |  |
| AB289451 | NtFLS_RT_F | GGCCTAAAAATCCTCCCTCCT |  |
|  | NtFLS_RT_R | TTCTCCACAACTTCTCGCAGC |  |
| 40644898 | NtHQT_RT_F | GTTGTGTTCACAGGCACACC |  |
|  | NtHQT_RT_R | CCACGGATTAAAGCCGATAA |  |
| GQ395697 | NtUFGT_RT_F2 | GAGTGCATTGGATGCCTTTT |  |
|  | NtUFGT_RT_R2 | CCAGCTCCATTAGGTCCTTG |  |
| FJ472647 | NtAN2_RT_F | GAAGAAAGGTGCATGGACTG |  |
|  | NtAN2_RT_R | TCTGCAGCTCTTTCTGCATC |  |
| AF154640 | NtActFw | GTGCTGAGCGTTTCCGTTGT |  |
|  | NtActRev | CTGCAGCTTCCATTCCAATCA |  |
