# Supplementary material for: Isolation and Characterization of the Flavonol Regulator CcMYB12 From the Globe Artichoke [Cynara cardunculus var. scolymus (L.) Fiori]
Source: Front Plant Sci. 2018 Jul 4;9:941. doi: 10.3389/fpls.2018.00941 (PMC6042477; doi:10.3389/fpls.2018.00941)
Supplement: Supplementary file 3 [file Image_1.PDF]

SUPPLEMENTARY FIGURE S1

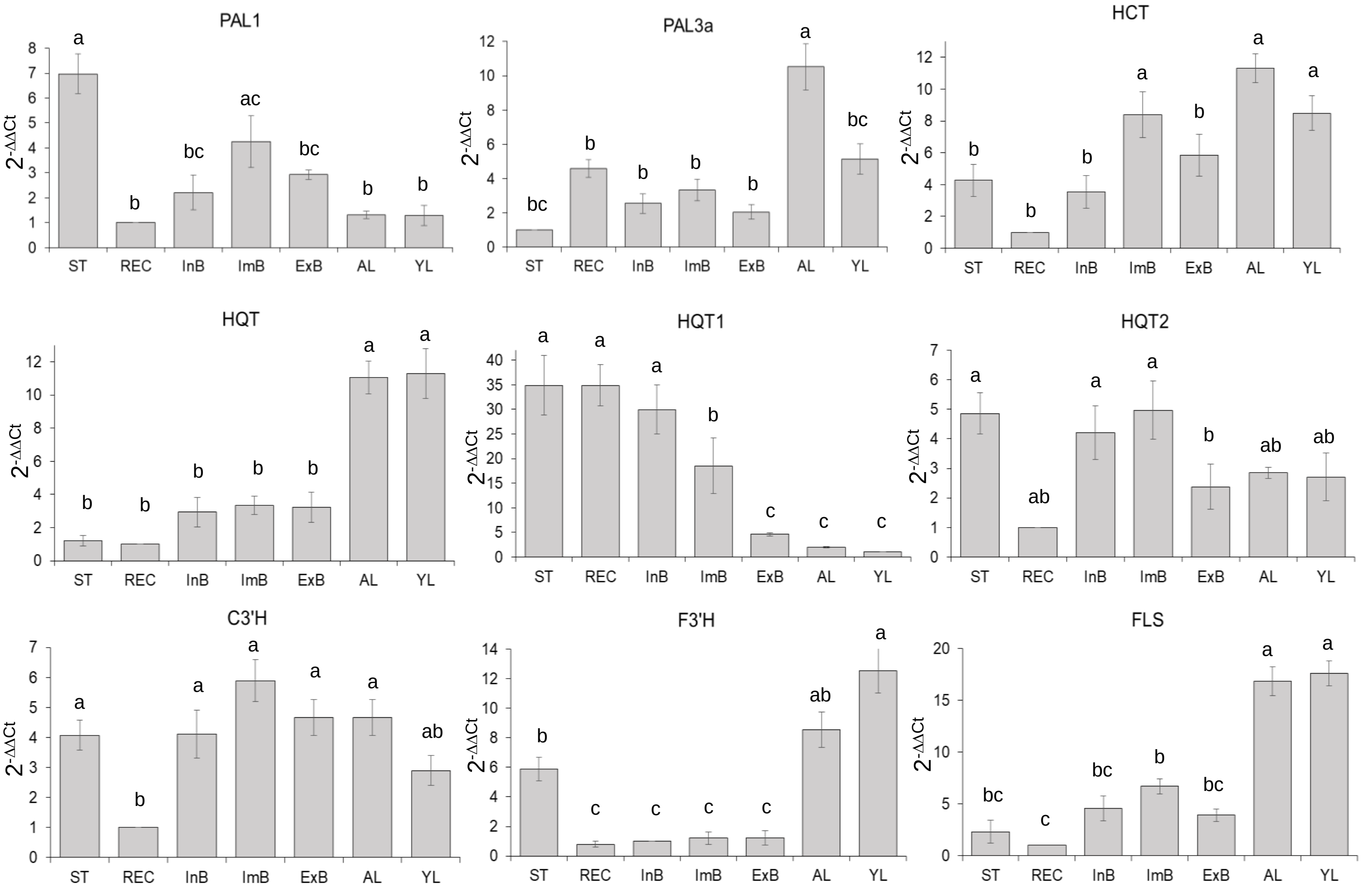

SUPPLEMENTARY FIGURE S1. Expression profiles of *CcMYB12* transcripts in artichoke organs. Expression levels of known artichoke biosynthetic genes (*HQT, C3'H, HCT, HQT1, HQT2, PAL1, PAL3* and *F3'H*) and of a putative *FLS* homologue in the following organs of artichoke: stem (St), receptacle (Rec), internal bracts (InB), intermediate bracts (ImB), external bracts (ExB), adult leaves (AL), young leaves (YL). Elongation Factor *EF1a* was employed as reference gene in all analyses. Values are calibrated to Rec for all genes, with the excepition of *HQT1* and of *F3’H,* where YL and InB were employed as calibrators, respectively. Values are means ± SD of three biological replicates. Bars with different letters are statistically different to each other according to one-way ANOVA Tukey test ($p < 0.05$).
