# Supplementary material for: Isolation and Characterization of the Flavonol Regulator CcMYB12 From the Globe Artichoke [Cynara cardunculus var. scolymus (L.) Fiori]
Source: Front Plant Sci. 2018 Jul 4;9:941. doi: 10.3389/fpls.2018.00941 (PMC6042477; doi:10.3389/fpls.2018.00941)
Supplement: Supplementary file 4 [file Image_2.PDF]

SUPPLEMENTARY FIGURE S2

ACII 5’ gatccttctccACCAACCcccttcacttcccg 3’
mACII 5’ gatccttctaaGAAGGAAaccttcacttcccg 3’

ACI 5’ gatcctttatacccACCTACCagacacg 3’
mACI 5’ gatcctttataaaaCAAGCAAagacacg 3’

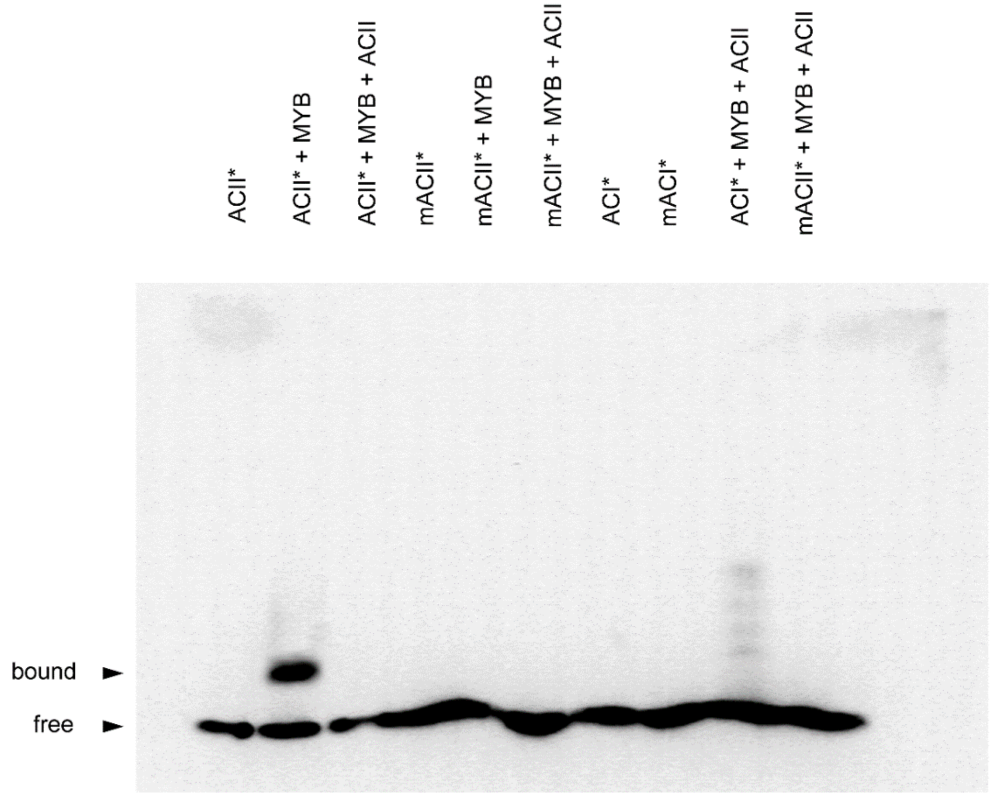

SUPPLEMENTARY FIGURE S2. Analyses of CcMYB12 protein binding to AC-elements ACII and ACI. EMSA output of the purified recombinant CcMYB12 binding to the AC element ACII or to its mutated counterpart mACII (left) and to ACI or mACI probe (right). The biotin-labeled free probes (ACII* and ACI*) without added protein is designed as control. Binding of recombinant CcMYB12 to biotin-labeled probes can be outcompeted by corresponding cold competitor. Nucleotide sequences of probes are shown on the top of the figure. The AC or mAC element is underlined.
