# Supplementary material for: Isolation and Characterization of the Flavonol Regulator CcMYB12 From the Globe Artichoke [Cynara cardunculus var. scolymus (L.) Fiori]
Source: Front Plant Sci. 2018 Jul 4;9:941. doi: 10.3389/fpls.2018.00941 (PMC6042477; doi:10.3389/fpls.2018.00941)
Supplement: Supplementary file 5 [file Image_3.PDF]

SUPPLEMENTARY FIGURE S3

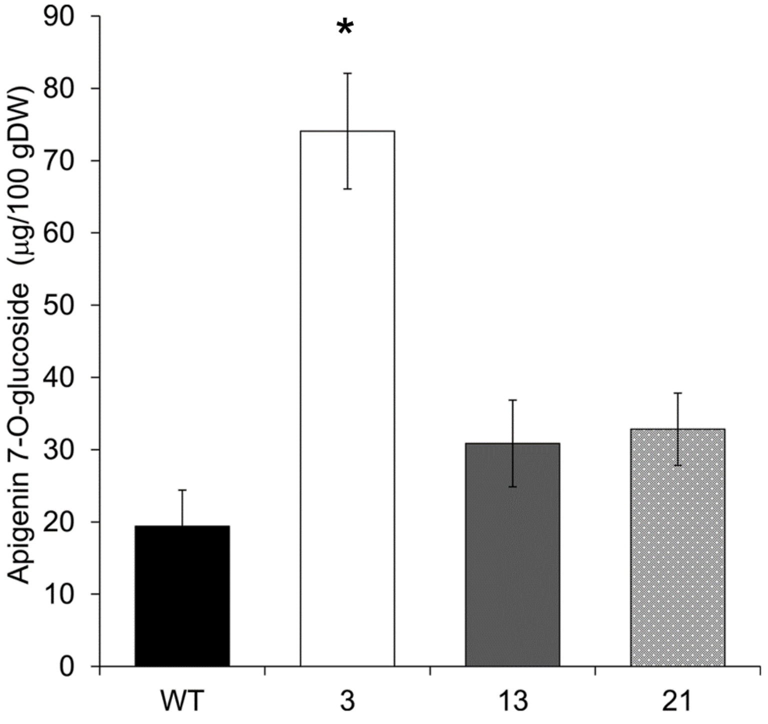

SUPPLEMENTARY FIGURE S3. **HPLC analyses of Apigenin7-O-glucoside in leaves of tobacco plants overexpressing CcMYB12 protein.** Leaves from wild type (WT) and T3 transgenic tobacco lines 3, 13 and 21 were tested. Error bars indicate the SD of the average of apigenin 7-O-glucoside determined as triplicates in three independent biological replicates. Asterisks indicate a statistical difference (*p-value < 0.05) between the means for WT and for tested transgenic samples, according to Student's t-test. DW, dry weight.
