# Supplementary material for: Isolation and Characterization of the Flavonol Regulator CcMYB12 From the Globe Artichoke [Cynara cardunculus var. scolymus (L.) Fiori]
Source: Front Plant Sci. 2018 Jul 4;9:941. doi: 10.3389/fpls.2018.00941 (PMC6042477; doi:10.3389/fpls.2018.00941)
Supplement: Supplementary file 6 [file Image_4.PDF]

SUPPLEMENTARY FIGURE S4

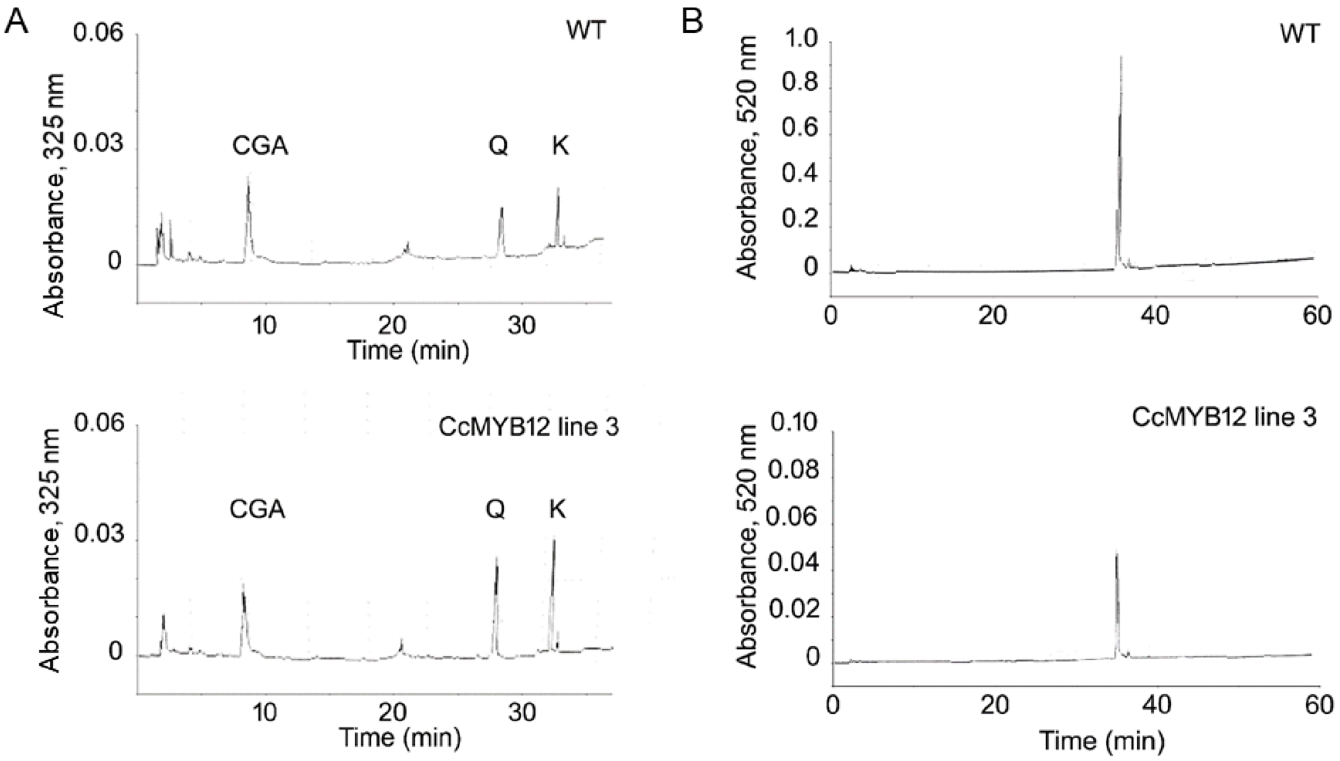

SUPPLEMENTARY FIGURE S4. HPLC profiles illustrating the accumulation of flavonoids and clorogenic acids in tobacco leaves and of anthocyanins in tobacco flowers. (A) Tobacco leaf methanolic extracts of WT (upper panel) and CcMYB12 line 3 (lower panel). (CGA) chlorogenic acid, (Q) quercetin-rutinoside, (K) kaempferol-rutinoside. Methanolic extract of leaf was separated with HPLC coupled with DAD detector at 325 nm, quantification with pure CGA, quercetin and kaempferol standards and identification with the help of Luo et al (2008). **(B)** Tobacco flower petals methanolic extracts of WT (upper panel) and CcMYB12 line 3 (lower panel). Cyanidin chloride was used as standard, detection at 520 nm, identification as in Luo et al (2007).
